# A Need to Shift from Glucose Centered to Cardio-Renal Centered Diabetes Care in Ethiopia

**DOI:** 10.4314/ejhs.v36i1.1

**Published:** 2026-01

**Authors:** Ermias Habte Gebremichael, Eyob Girma Abera

**Affiliations:** 1 Department of Internal Medicine

The global burden of type 2 diabetes mellitus is rising rapidly, with the greatest proportional increases occurring in low- and middle-income countries. In Ethiopia, this growth is particularly concerning. According to the International Diabetes Federation, approximately 2.3 million Ethiopian adults aged 20–79 years were living with diabetes in 2024, with millions more undiagnosed or living with impaired glucose tolerance (1). Ethiopia has a largely young population, yet urbanization, changing diets, and reduced physical activity are contributing to a growing incidence of type 2 diabetes among adults in the workforce.

The clinical management of type 2 diabetes is undergoing a fundamental transformation globally. For decades, treatment paradigms were anchored primarily in glycemic control, with fasting plasma glucose and glycated hemoglobin (HbA_1_c) serving as the principal therapeutic targets. While tight glycemic control remains important for reducing microvascular complications, a growing body of evidence demonstrates that cardiovascular disease and chronic kidney disease are the dominant drivers of morbidity and mortality in people with type 2 diabetes. This recognition has catalyzed a shift from a purely glucose-centered approach toward a cardio-renal–centered model of care that prioritizes organ protection alongside glycemic management (2).

Large cardiovascular outcome trials have shown that newer glucose-lowering therapies, particularly sodium-glucose cotransporter-2 inhibitors (SGLT2 inhibitors) and glucagon-like peptide-1 receptor agonists (GLP-1 receptor agonists), reduce major adverse cardiovascular events, hospitalization for heart failure, and progression of kidney disease, often independent of their glucose-lowering effects. This evidence has reshaped international treatment guidelines, which now recommend selecting therapy based on an individual's cardiovascular and renal risk profile rather than HbA_1_c alone. In many high-income settings, these agents are increasingly used early in the treatment course for patients with established cardiovascular disease, heart failure, or chronic kidney disease (3).

In Ethiopia, however, diabetes care remains largely anchored in a glucocentric paradigm. Metformin, sulfonylureas, and insulin constitute the backbone of therapy in most public health facilities, reflecting historical practice patterns and the realities of drug availability and affordability. While these agents are effective for lowering blood glucose, they do not confer the same level of cardio-renal protection demonstrated by SGLT2 inhibitors and GLP-1 receptor agonists (2). As a result, many Ethiopian patients may achieve modest glycemic control yet remain exposed to a high risk of cardiovascular and renal complications.

Limited access to newer cardio-renal protective therapies is a central barrier. SGLT2 inhibitors and GLP-1 receptor agonists are either unavailable in many public facilities or unaffordable for most patients when available in the private sector. Health systems research from Ethiopia highlights frequent stock-outs of essential medicines, high out-of-pocket expenditures, and weak supply chains, all of which constrain the adoption of newer therapies (4). Without deliberate policy action, the global shift toward cardio-renal centered care risks becoming another example of therapeutic inequity.

The implications are substantial. Cardiovascular disease is already a leading cause of death among people with diabetes worldwide, and kidney failure imposes catastrophic costs on families and health systems. Continuing to focus primarily on glucose targets, while neglecting therapies that modify these outcomes, will likely perpetuate avoidable morbidity and premature mortality in Ethiopia.

A pragmatic transition toward cardio-renal centered diabetes care in Ethiopia does not require abandoning glycemic control. Rather, it calls for integrating glucose management within a broader framework that prioritizes cardiovascular and renal risk reduction. Updating national treatment guidelines, expanding essential medicine lists to include proven cardio-renal protective agents, strengthening procurement and supply systems, and building clinician capacity for risk-based treatment selection are achievable steps. The global narrative of diabetes care has moved beyond glucose alone; Ethiopia now faces a critical opportunity to ensure that its national response keeps pace with this evolution.
